# Does clozapine really affect bone mineral density? An experimental study

**DOI:** 10.1186/s13018-021-02695-w

**Published:** 2021-09-15

**Authors:** Bahattin Kerem Aydin, Selim Safali, Memduha Aydin, Umran Egilmez, Hakan Cebeci, Murat Çelik, Ummuhan Abdulrezzak

**Affiliations:** 1 Faculty of Medicine, Department of Orthopedics and Traumatology, Selcuk University, Alaeddin Keykubat Campus, 42100 Selcuklu, Konya, Turkey; 2 Faculty of Medicine, Department of Psychiatry, Selcuk University, Konya, Turkey; 3 Faculty of Medicine, Department of Radiology, Selcuk University, Konya, Turkey; 4 Faculty of Medicine, Department of Pathology, Selcuk University, Konya, Turkey; 5 Faculty of Medicine, Department of Nuclear Medicine, Erciyes University, Kayseri, Turkey

**Keywords:** Osteoporosis, Schizophrenia, Rat, Bone mineral density, Clozapine

## Abstract

**Purpose:**

The aim of this study was to investigate the effect of clozapine use on bone tissue by applying computerized tomography, dual-energy X-ray absorptiometry, and histological and biomechanical analyses in an experimental rat model.

**Methods:**

Sixteen female Wistar Albino rats were included in this study. These animals were divided into two groups: the control group and the clozapine group. The animals in the clozapine group received 10 mg/kg clozapine, and the animals in the control group received tap water by oral gavage daily for 28 days. After sacrification, the femurs of the rats were used for radiologic, histologic, dual-energy X-ray absorptiometry, and biomechanical evaluations.

**Results:**

Although the mean values of the clozapine group were higher in terms of histological, bone mineral density, and biomechanical evaluations, the statistical analyses were not significantly different.

**Conclusion:**

Clozapine use did not affect bone density in the rats. Clozapine can be the preferred treatment for patients with schizophrenia to avoid osteoporosis. It will be necessary to conduct further long-term follow-up and controlled studies in animals and humans to confirm these findings.

## Introduction

Schizophrenia is a significant psychiatric disorder with a prevalence of 15/100,000 worldwide [1]. As it is a chronic mental disorder, long-term medical treatment with antipsychotic drugs is often needed.

Routine medical treatment of schizophrenia consists of “typical antipsychotics” such as haloperidol. Haloperidol primarily inhibits dopaminergic pathways and causes extrapyramidal side effects and postural hypotension [2]. Another important side effect is hyperprolactinemia.

To decrease these side effects, new generation antipsychotics are used for schizophrenia treatment. New generation antipsychotics, or “atypical antipsychotics” (such as clozapine, olanzapine, quetiapine), are also called prolactin-sparing agents. These drugs have less anti-dopaminergic activity, have become widely used because of their greater antipsychotic efficacy, and have fewer extrapyramidal side effects [2, 3].

Clozapine may be the most important agent among these atypical antipsychotic drugs as it is the most efficacious and is considered the “last-line” treatment for schizophrenia. For this reason, it is difficult to change clozapine treatment to other antipsychotics after it is instituted [4]. Therefore, the adverse effects of clozapine are important as it may be used lifelong.

Osteoporosis is also a significant issue in patients with schizophrenia. Osteoporosis and secondary fractures contribute to high morbidity and mortality especially in patients with schizophrenia. Secondary hyperprolactinemia due to antipsychotic use is known to be a cause of osteoporosis [2, 5–7]. This effect can be a reason for using clozapine instead of haloperidol.

There are a limited number of publications on clozapine’s effect on bone mineral density (BMD), and the results are inconclusive. Some of the studies used only Dual-energy X-ray absorptiometry (DEXA), and some examined BMD using both DEXA and histology. Based on our current knowledge, no studies in the English literature have assessed clozapine’s effect on BMD using computed tomography (CT), DEXA, histology, and biomechanical tests in the same study. In this study, we aimed to investigate the effect of clozapine use on bone tissue by using these four evaluation methods in rats.

## Materials and methods

The study was designed according to the Helsinki Declaration. We were granted permission from the Selcuk (permission number 2019-78) before starting the study.

Sixteen female Wistar Albino rats aged 3–4 months and weighing 300–400 gr were included in this study. These animals were divided into two groups: the control group and the clozapine group.

The dosage of clozapine was planned according to previous publications [3]. Clozapine (Leponex, MEDA Pharma, Istanbul, Turkey) was administered 10 mg/kg by oral gavage daily for 28 days. The same volume of tap water was administered to the control group by oral gavage daily for 28 days. After 28 days, all the rats were sacrificed using high-dose ketamine. After radiologic evaluations, the right femurs were used for histological examination, and the left femurs were used for biomechanical evaluations.

All the femora were transferred to the Selcuk Radiology Department for CT evaluation. The CT images were examined by an experienced radiologist who was blind to the study. All CT examinations were performed on a 16-slice CT (Somatom Scope, Siemens, Germany). The following technical parameters were used: tube voltage: 130 kV; tube current modulation: 22 mAs; spiral pitch factor: 0.95; collimation width: 0.6. DICOM data were transferred onto a PACS Workstation. Regions of interest between 2 and 10 mm^2^ were localized to the cortex and medulla of the bone. Mean, maximum, and minimum Hounsfield Unit values were noted.

After radiologic imaging, all the femurs were transferred to the Nuclear Medicine Department Selcuk, Selcuk DEXA evaluation. Rat BMD (g/cm^2^) was measured using the mean measurements of DEXA on a Hologic Horizon Wi device (Hologic, MA, USA). All the specimens were scanned by the same operator.

For the histological evaluation, all the femurs were fixed in a 10% formalin solution for 2 days. A 10% acetic acid solution was used for decalcification over the course of 1 day. Next, the specimens were embedded in paraffin, and 4-μm-thick serial sections were taken. Hematoxylin and eosin staining was performed. Histological evaluation was conducted using an Olympus BX43 light microscope. Photographs of the specimens were taken under × 600. After counting the osteocytes and osteoblasts, the ratio of osteocytes to osteoblasts was calculated individually for all specimens.

Three-point bending tests were used for the biomechanical evaluation. Eight femurs from each group were examined in the Biomechanical Laboratory Selcuk using the Elista TST 2500 material testing device. The femurs were mounted with mini-clamps on the testing machine. The clamps gripped each specimen at the distal and proximal metaphyseal parts of the bone. For each bone, these supports were placed individually so a clamp was under the trochanter major and a clamp was under the distal femur. To prevent twisting of the position during loading, the intercondylar fossa of each femur was gently but tightly pressed between the pliers attached to the testing device.

Before the actual testing, a small stabilizing preload (10 N) was applied on the medial surface of the femur at a rate of 0.1 mm/s using a steel cross-bar fixture (a plate with rounded edges with a 10-mm diameter). The plate was oriented perpendicularly to the long axis of the bone and at the midpoint between the lower ends. The bending load was applied at a rate of 1.0 mm/s until failure of the specimen. The breaking load of the femoral midshaft was determined from load-deformation curves, and the maximum loads just before fractures were used for the statistical analysis.

Statistical analyses were performed using IBM SPSS version 20 (IBM Corp., Armonk, NY, USA). The Mann–Whitney *U* test was used for analyzing the radiological, histological, and biomechanical results to evaluate the differences between the two groups. Risk values were calculated using a 95% confidence interval. A *p* value < 0.05 was considered to be significant.

## Results

After sacrification, 16 femurs from the control group and 16 femurs from the clozapine group were included. All 32 femurs were used for the radiologic and BMD evaluations. Eight femurs from each group were used for the histological examination, and the remaining eight femurs from each group were used for the biomechanical tests. All the results of the groups are shown in Table 1 using mean values.

**Table 1:** The mean results of bone mineral density measurements according to histological, biomechanical, radiological, and DEXA evaluations

	Control Group Mean value	Clozapine Mean value	***p value***
**Histological evaluation** (osteocyte:osteoblast ratio)	2.468	2.257	0.111
**Biomechanical evaluation** (three-point bending)	195.3	213.4	0.103
**Radiological evaluation** (Density of cortex and medulla) (Hounsfield units)	Cortex 902 Medulla 291.8	Cortex 1001 Medulla 285.8	0.128 0.861
**DEXA measurements** (BMD g/cm^2^)	0.2127	0.2102	0.662

### Radiological results

According to the CT evaluation, neither the cortex nor medulla measurements of the two groups revealed any significant difference.

### BMD results

The mean DEXA measurement was higher in the clozapine group. However, the results were not significant.

### Histological results

Although the clozapine group showed a better mean value, there was no significant difference between the groups.

### Biomechanical results

According to the three-point bending results, the mean load needed to fracture the femurs was higher in the clozapine group. However, no significant difference was detected between the two groups.

## Discussion

The effect of clozapine on bone density was evaluated in this experimental study. Up to now, this is the first study investigating this topic by using radiologic, DEXA, histologic, and biomechanical examinations at the same time. According to our results, clozapine has no adverse effects on bone density.

The effect of clozapine on bone density has been studied before [2, 8–16]. However, the results of these studies were not consistent. Some of the studies concluded that clozapine decreases bone density [2, 12], and others concluded that clozapine has a positive effect on bone density [8, 9, 11, 16]. A few studies concluded that clozapine has no effect on bone density [13, 15]. All the previous studies used different investigation techniques such as radiological, DEXA, or histological methods. Also, some of the previous studies used experimental models, and others were retrospective clinical studies.

BMD is an important issue for elderly people. Common clinical cases are hip, spine, and radius fractures. Chronic medications can potentially affect BMD. Patients with schizophrenia have to use their medications regularly for their entire lifetime. Haloperidol, known as the first-line treatment for schizophrenia, has unwanted effects which include increases in prolactin levels and extrapyramidal symptoms. These side effects are important issues after cessation of haloperidol. Clozapine, a new generation antipsychotic drug, has become the gold standard in schizophrenia treatment. Clozapine has limited side effects compared to haloperidol. However, its major side effect is agranulocytosis, which can cause a decrease in white blood cells. Similarly, haloperidol’s effect on prolactin is an important issue. Haloperidol can increase prolactin levels as much as in menopause. Accordingly, the risk of low bone density is secondary to this effect of haloperidol. Clozapine is also preferred compared to haloperidol as its effect on prolactin levels is limited.

The most commonly used determining factors for BMD are radiologic, histologic, biomechanical, and biochemical parameters. Radiologic parameters can be examined by CT and DEXA devices. A radiological investigation was applied to all 32 femurs of the rats in this study. For the CT evaluation, the cortex and medulla measurements used Hounsfield units. Additionally, DEXA measurements were applied to all the femurs. There was no significant difference between the control and clozapine groups in this study.

A clinical retrospective study from Taiwan [8] concluded that long-term clozapine treatment may be protective for BMD compared to treatment with non-clozapine antipsychotics. However, only DEXA measurements of the patients were investigated in this study.

Another paper [9] compared the effects of prolactin-sparing and prolactin-raising drugs on BMD in female patients, and it was found that clozapine treatment was beneficial for BMD compared to prolactin-raising antipsychotic treatment in women with chronic schizophrenia. The study also suggested that clozapine’s bone density-protecting effect is dose-related with higher doses associated with denser BMD. The study used a retrospective clinical design and compared DEXA measurements, age, body mass index, and blood parameters including thyroid stimulating hormone, alkaline phosphatase, prolactin, and calcium.

The negative effect of clozapine on BMD was also reported. Researchers studied both clozapine’s and haloperidol’s effects using an experimental rat model study. They used DEXA and micro-CT measurements for BMD and the number of osteoblast and osteoclasts [2]. According to their study, long-term administration of clozapine may negatively affect bone health, and clinical studies to investigate this possibility are warranted. The authors also commented that clozapine, but not haloperidol, exerts adverse skeletal effects, and this effect may be attributable to direct actions to reduce osteoblast growth and function. This finding is not consistent with our results. Although they used micro-CT and DEXA measurements, they did not use any histological or biomechanical evaluations. It is interesting that this study’s results are not consistent with those of our study and other clinical studies.

According to another clinical study [12], a decrease in BMD was detected in male patients with schizophrenia who had taken clozapine for a long period of time. They used DEXA and biochemical parameters. The results of this paper were not consistent with those of previous clinical studies; it was a cross-sectional retrospective study without a control group. The authors also indicated that other factors such as exposure to sunlight, calcium intake, and amount of exercise may be important issues in patients with schizophrenia.

In another paper, the researchers concluded that clozapine use is beneficial for BMD compared to prolactin-raising antipsychotics in women with chronic schizophrenia. They also noted that the risk factors associated with low BMD are different between men and women [11]. According to a review consisting of five studies [13], switching to atypical antipsychotics reduces hyperprolactinemia and improves hypogonadism and related clinical symptoms. Prolactin levels and DEXA measurements were primarily used in these five studies.

A prospective clinical study [15] compared the effects of two generations of antipsychotics (both typical and atypical) on BMD and its association with prolactin at baseline and 12 months after treatment. According to their results, no significant change was found in patients taking atypical antipsychotics in contrast to patients taking typical antipsychotics. The study also commented that the increase in PRL level is an important risk factor leading to osteoporosis after long-term use of typical antipsychotics.

According to the results of another clinical study [16], patients with schizophrenia who had osteoporosis were female and older, displayed lower weight and body mass index, or were treated with non-clozapine antipsychotics. They concluded that clozapine treatment may be beneficial for low BMD, and the treating psychiatrist could consider switching to clozapine or another lower-risk medication in patients who already are at high risk for osteoporosis.

The findings of the previous studies suggest that schizophrenic patients taking “typical antipsychotics” (such as haloperidol) are characterized by a significant lower bone mineral density than patients taking “atypical antipsychotics” (such as clozapine, olanzapine and quetiapine) [17]. This effect can be a reason for using clozapine instead of haloperidol. The cross-sectional study that was carried out with the schizophrenic patients who have undertaken the monotherapy with “atypical antipsychotics” olanzapine, clozapine, and risperidone, for at least 1 year found no significant relationship between hyperprolactinemia and the decreased BMD [12].

There are several limitations that should be considered in evaluating the present study. First, the number of the rats was limited, so the capacity to identify a small effect was limited. Second, all the rats were female, so a comparison between genders could not be conducted. The follow-up time could have been longer, as this study’s aim was to investigate the effect of clozapine which is often used for an entire lifetime. Another limitation is the lack of biochemical markers that can be used for investigating bone tissue.

In conclusion, clozapine use did not affect bone density in the rats according to the CT, DEXA, histologic, and biomechanical evaluations. Clozapine can be the preferred treatment for patients with schizophrenia to avoid osteoporosis. It will be necessary to conduct further long-term follow-up and controlled studies in animals and humans to confirm these findings.

## Data Availability

The histological specimens, radiologic images, and Dexa images are available as they are stored in the archives of the clinic.

## Abbreviations

BMD: Bone mineral density
DEXA: Dual-energy X-ray absorptiometry
CT: Computed tomography

## References

[1] McGrath J, Saha S, Welham J, El Saadi O, MacCauley C, Chant D (2004). A systematic review of the incidence of schizophrenia: the distribution of rates and the influence of sex, urbanicity, migrant status and methodology. BMC Med.

[2] Costa JL, Smith G, Watson M, Lin JM, Callon K, Gamble G (2010). The atypical anti-psychotic clozapine decreases bone mass in rats in vivo. Schizophr Res.

[3] Freedman R (2003). Schizophrenia. N Engl J Med.

[4] Correll CU, Rummel-Kluge C, Corves C, Kane JM, Leucht S (2009). Antipsychotic combinations vs monotherapy in schizophrenia: a meta-analysis of randomized controlled trials. Schizophr Bull.

[5] Halbreich U, Rojansky N, Palter S, Hreshchyshyn M, Kreeger J, Bakhai Y (1995). Decreased bone mineral density in medicated psychiatric patients. Psychosom Med.

[6] Howard L, Kirkwood G, Leese M (2007). Risk of hip fracture in patients with a history of schizophrenia. Br J Psychiatry.

[7] Hugenholtz GW, Heerdink ER, van Staa TP, Nolen WA, Egberts AC (2005). Risk of hip/femur fractures in patients using antipsychotics. Bone..

[8] Lin CH, Lin CY, Wang HS, Lane HY (2019). Long-term use of clozapine is protective for bone density in patients with schizophrenia. Sci Rep.

[9] Lin CH, Huang KH, Chang YC, Huang YC, Hsu WC, Lin CY (2012). Clozapine protects bone mineral density in female patients with schizophrenia. Int J Neuropsychopharmacol.

[10] Lobach AR, Uetrecht J (2014). Clozapine promotes the proliferation of granulocyte progenitors in the bone marrow leading to increased granulopoiesis and neutrophilia in rats. Chem Res Toxicol.

[11] Chen CY, Lane HY, Lin CH (2016). Effects of antipsychotics on bone mineral density in patients with schizophrenia: gender differences. Clin Psychopharmacol Neurosci.

[12] Lee TY, Chung MY, Chung HK, Choi JH, Kim TY, So HS (2010). Bone density in chronic schizophrenia with long-term antipsychotic treatment: preliminary study. Psychiatry Investig.

[13] O'Keane V (2008). Antipsychotic-induced hyperprolactinaemia, hypogonadism and osteoporosis in the treatment of schizophrenia. J Psychopharmacol.

[14] Schjerning O, Lykkegaard S, Damkier P, Nielsen J (2015). Possible drug-drug interaction between pregabalin and clozapine in patients with schizophrenia: clinical perspectives. Pharmacopsychiatry..

[15] Wang M, Hou R, Jian J, Mi G, Qiu H, Cao B (2014). Effects of antipsychotics on bone mineral density and prolactin levels in patients with schizophrenia: a 12-month prospective study. Hum Psychopharmacol.

[16] Cui J, Liu H, Shao J, Xu DM, Wang Y, Fei Z (2018). Prevalence, risk factors and clinical characteristics of osteoporosis in Chinese inpatients with schizophrenia. Schizophr Res.

[17] Bilici M, Cakirbay H, Guler M, Tosun M, Ulgen M, Tan U (2002). Classical and atypical neuroleptics, and bone mineral density, in patients with schizophrenia. Int J Neurosci.

